# School-based interventions targeting stigma of mental illness: systematic review

**DOI:** 10.1192/pb.bp.112.041723

**Published:** 2014-08

**Authors:** Catriona Mellor

**Affiliations:** 1 Oxford Health NHS Foundation Trust, Oxford, UK

## Abstract

**Aims and method** To systematically review the published literature on the effectiveness of classroom-based interventions to tackle the stigma of mental illness in young people, and to identify any consistent elements within successful programmes.

**Results** Seventeen studies were included in the analysis. A minority of studies reported a positive impact on stigma or knowledge outcomes at follow-up and there were considerable methodological shortcomings in the studies reviewed. These interventions varied substanitally in content and delivery. It was not possible to use this data to draw out what aspects make a successful intervention. There is currently no strong evidence to support previous conclusions that these types of intervention work for children and adolescents.

**Clinical implications** When anti-stigma interventions for young people are rolled out in the future, it is important that the programme design and method of delivery have evidence to prove their effectiveness, and that the audience and setting are the most appropriate to target. There is a current lack of strong evidence to inform this.

It is estimated that in high-income countries 5-20% of children and adolescents require mental health services and, in Europe, provision of services to those in need can be as low as 20%.^1^ There is growing evidence that barriers to seeking help and achieving recovery for mental health problems include the stigma around mental illness,^2,3^ and that stigmatising attitudes start young.^4^ Stigmatising attitudes to mental illness are widespread.^5^ There is national and international recognition that this issue should be prioritised.^6,7^ There is mixed evidence as to whether national initiatives can change such attitudes.^8-11^ Attempts to research and implement school-based mental health promotion in the UK have largely focused on making the whole-school environment more emotionally aware.^12-15^ However, there are also many initiatives, in the UK and other countries, taking anti-stigma programmes into the classroom (for example Rethink, Royal College of Psychiatrists). One review concluded that educational interventions in schools provide positive outcomes on pupils’ attitudes to mental illness,^16^ a belief that seems widespread in the literature. However, it reports positive findings of the studies reviewed without clearly describing the quality of the studies, making the findings difficult to interpret. Its conclusions are considerably more optimistic than an earlier review that questioned the reliability and validity of all studies reviewed.^17^ However, there have been many published studies since 2006 (the limit of Schachter’s *et al*’s meticulous report^17^), which may explain the discrepancy. This systematic review addresses two specific questions: (a) what current evidence is there to justify the growing optimism as to the effectiveness of school-based anti-stigma programmes and (b) what evidence is there to inform future successful programme design?

## Method

### Inclusion criteria

The types of studies included (using Cochrane Effective Practice and Organisation of Care (EPOC) group definitions) were randomised controlled trials (RCTs), cluster RCTs, non-randomised controlled trials (NRTs), or controlled before-after studies (CBA). Participants were children or adolescents attending primary or secondary school. School-based interventions targeting attitudes and stigma about mental illness were included. Studies were included if they measured outcomes of: knowledge/beliefs and attitudes towards mental illness, behavioural intentions, stigmatising behaviour or affect. The analysis of help-seeking outcomes is not covered in this review, because help-seeking is not directly associated with stigmatising attitudes/behaviour. Level of knowledge is also not directly associated with stigmatising attitudes but these outcomes are included as many of the ‘knowledge’ measures contain some belief and attitude statements. Known reliability/validity of the instruments was not an inclusion criterion, but will be commented on within the results.

### Search methods and study selection

The following search engines were used: Medline, CINAHL and PsycINFO (1990-2013, articles in English) on 12 June 2013, using the keywords (stigma^*^ OR attitude^*^ OR awareness) AND (school or adolesc^*^) AND (educat^*^ OR train^*^ OR program^*^) AND (mental OR schizophreni^*^ OR psychiatri^*^). The references lists cited in relevant reviews were also checked.^16-21^ Studies were selected for inclusion by screening titles, abstracts and when necessary full texts, against the inclusion criteria.

### Data extraction and critical appraisal

A data-extraction form based on the Cochrane EPOC group’s data-collection checklist was used to record details about study characteristics, intervention design, outcome measures and results. Following this process the group’s recently updated ‘suggested risk of bias criteria for EPOC reviews’^22^ was used to make judgements on the risk of bias (high, low or unclear) in each study in each of the domains suggested by the document. The domains assessed were: allocation sequence generation and concealment, baseline outcome measures and characteristics, comparison of site profiles (if applicable), protection against contamination, masking, completeness of outcome data, and outcome reporting (were data for each outcome, group and time point fully presented). In addition to this, the reliability and validity of the instruments used, as documented in the study reports, was noted.

### Data synthesis

The review looked at the intervention effect of each study by comparing before and after outcome scores in the intervention and control groups. First, studies that provided follow-up data (rather than simply immediate post-test data) were reviewed. Of these, studies that reported a positive result (a statistically significant, *P*<0.05, change in any outcome measure compared with control) after the intervention were selected. These studies were reviewed for study quality, as judged by study design and risk of bias criteria. Studies with positive results at immediate post-intervention only were then reviewed for study quality. Positive results based on the use of specifically developed outcome measures with low reliability were excluded. To answer the second review question the intervention design features (such as duration, contact or non-contact, delivery) of those studies showing positive results were tabulated and compared.

**Fig 1:**
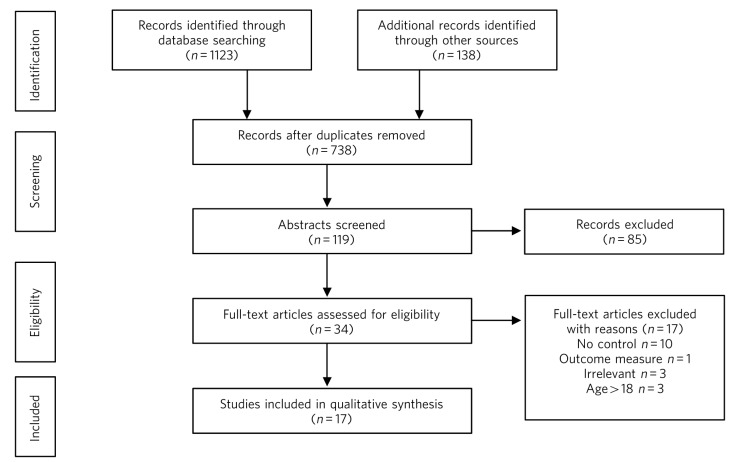
Selection of studies using Preferred Reporting Items for Systematic reviews and Meta-Analyses (PRISMA).

## Results

Of the 1261 studies identified in the initial search, 17 met the above criteria (Fig. 1).^23^

### Intervention and study characteristics

The interventions varied in content and delivery methods (online Table DS1). Nine were education-only,^24-32^ whereas eight had indirect^33,34^ or direct^35-40^ contact with someone with lived experience. Fifteen studies targeted secondary-school pupils, two targeted primary school pupils.^28,31^ One included a few individuals over 18.^35^ The duration ranged from one-off interventions lasting 30-120 min to multiple sessions over a period of up to 4 months. The focus of the interventions was mental illness in 11 studies, schizophrenia in 3 and depression in 3. Five studies investigated the impact of already established interventions.^30,36-38,40^ The number of participants varied from 40 to 616. The follow-up time ranged from immediately post intervention only, up to 12 months. The outcome measures were secondary outcomes in two studies,^26,27^ which are shown at the end of Table DS1. One study was an RCT.^34^ Five studies were cluster RCTs, two using cluster randomisation at the school level,^24,28^ three using cluster randomisation at the class/year level within selected schools.^32,33,40^ Four studies were NRTs, six were CBA trials. It is unclear whether one study was an NRT or CBA.^38^

The comparison groups, other than Chan *et al*’s,^33^ which compared three intervention conditions, had normal lessons (no intervention) in 14 of the studies, a talk about healthy living from external speakers in 1^32^ and a video presentation about smoking in another.^34^ The vast majority therefore did not control for the effect of a novel programme, in many cases with outside speakers.

#### Outcome measures

Table DS1 shows all outcome measures used within the studies. Results from two additional scales were excluded as irrelevant to the review question (the Self-Efficacy Scale^38^ and the Strengths and Difficulties Questionnaire^29^). Of the remaining 31 outcome measures used (and reviewed here), most measures were ‘stigma’ measures: attitudes, behavioural intentions and in one study an affect measure.^40^ In addition, several studies tested factual knowledge gained. No studies measured actual behaviour. All measurements were self-report Yes/No, True/False or Likert-style questionnaires, except for the Implicit Association Test (IAT),^34^ where participants categorise words as quickly as possible.

For 14 of the instruments reliability was reported as good, in all but one of these cases the studies chose to report internal consistency (Cronbach’s alpha) to back up that claim. The instruments’ validity was usually not mentioned in the report, although some studies used previously well-tested instruments.

A total of 13 of the instruments were designed for the intervention or study; 6 of these had poor (or untested) reliability, casting doubt also on their validity^24,29-31,37,40^ and therefore on the results that they provide. These six were all knowledge measures. The other seven were piloted and/or internally consistent.^30,31,33,36^

#### Study quality

Details of study quality are provided in Table 1. Only one study, a cluster RCT, adequately described randomisation and allocation concealment.^24^ Baseline outcome measures and baseline group characteristics were clearly compared and similar in nine (in addition, one study showed similarity in one but not the other outcome^37^) and six studies respectively. Four studies used different sites as their control and intervention groups and none of these studies clearly compared (with measures of significance) the sites’ profiles. These four studies only were able to clearly protect against contamination. Due to using self-report questionnaires none of the outcome measures were masked or objective (the IAT is ‘self-report’ but aims to assess automatic memory associations and therefore is less open to bias^34^). There was the potential of attrition bias being introduced because of incomplete data in 13 studies. Most of the studies did not omit important outcome data in their reports. Four studies mention a power calculation. One was underpowered,^40^ the other three report having sufficient sample size.^26,27,29^

Various methods were used to enhance consistency of delivery. In two studies the presenters were trained and sessions monitored for fidelity^29,40^ and two interventions used a computer program.^26,27^ Five others mention training the presenters,^24,33,34,37,38^ the remaining eight provided material for the presenters to follow.

### Intervention effects

To answer the first review question it is helpful to look at whether the studies with positive changes in stigma (and knowledge) outcomes are of high enough quality to give confidence in their findings. The final two rows of Table 1 show which studies reported statistically significant results at follow-up (for knowledge and stigma outcomes). Results of each outcome measure are tabulated as either reporting a significant positive change (a tick) or no significant change (a cross).

Table 2 gives an overview of the results reported in the studies at post-test and follow-up, and indicates whether the outcomes measured changed significantly (a tick) or not (a cross). Results from the six outcome measures developed specifically for the interventions they were testing, with reliability not measured (or α < 0.7), are not included in this section. Table 2 shows which outcome measures this applies to (represented by **/**). For two studies, where this involved the only instrument used,^24,29^ there are therefore limited conclusions that can be drawn here despite the fact that they did otherwise have relatively good methodology, according to the risk of bias table.

#### Studies with positive results at follow-up

Twelve studies collected information at follow-up. Of these, seven studies showed some statistically significant positive changes at follow-up,^25,30-33,35,36^ and these are summarised here. All were at high risk of selection bias except for the two cluster RCTs, which did not, however, have a clearly described method of randomisation. All had high-risk levels of attrition or an unclear description of individuals who dropped out, except for Economou *et al*.^32^

Economou *et al*’s^32^ cluster RCT compared change in mean score per item on their belief/attitude questionnaire and reported that 8/10 items were answered significantly better at follow-up than baseline in the intervention group. They report no significant change in the control group but do not present these data. There was no significant improvement in social distance scores at follow-up.^32^ Chan *et al*’s^33^ cluster RCT showed significant positive change in knowledge and social distance but not stigma at follow-up. This study discarded 35% of their data (because of absenteeism or returning incomplete measures) and it was not clear from which group(s) these missing data were from.

Ventieri *et al*’s^31^ study in a primary school used Schulze *et al*’s^36^ social distance scale and a novel instrument to test ‘benevolence’ and ‘unkindliness’, piloted on a group of pre-adolescents and tested for reliability. The intervention group showed positive change compared with the controls in all three measures. Schools were invited into the study based on assignment (to control or intervention).^31^ In Wahl *et al*’s^30^ study, mean total score in knowledge, attitudes and social distance (on scales developed for the study) improved slightly but significantly. Only 47% of eligible pupils were included in the analysis (those who took part in the three-session programme and completed all questionnaires).

**Table 1:** Study design, quality and outcomes

	Saporito *et al* (2011)^34^	Economou *et al* (2011)^32^	Pinto-Foltz *et al* (2011)^40^	Chan *et al* (2009)^33^	Pitre *et al* (2007)^28^	Rahman *et al* (1998)^24^	Ventieri *et al* (2011)^31^	Wahl *et al* (2011)^30^	Robinson *et al* (2010)^39^	Naylor *et al* (2009)^29^	Conrad *et al* (2009)^38^	O’Kearney (2009)^27^	O’Kearney *et al* (2006)^26^	Rickwood *et al* (2004)^37^	Schulze *et al* (2003)^36^	Ng & Chan (2002)^35^,^d^	Esters *et al* (1998)^25^
Design	RCT	Cl-RCT	Cl-RCT	Cl-RCT	Cl-RCT	Cl-RCT	NRT	NRT	NRT	NRT	? NRT	CBA	CBA	CBA	CBA	CBA	CBA
																	
Intervention^a^	I, Ed	Ed	C, Ed	I, Ed	Ed	Ed	Ed	Ed	C, Ed	Ed	C, Ed	Ed	Ed	C, Ed	C, Ed	C, Ed	Ed
																	
Duration^b^	1	1	1	1	?	3	2	2	1	3	2	3	3	1	2	3	2
																	
Risk of bias^c^																	
1	?	?	?	?	?	✓	˟	˟	˟	˟	˟	˟	˟	˟	˟	˟	˟
2	?	?	?	?	?	✓	˟	?	?	?	?	˟	˟	˟	˟	˟	˟
3	?	✓	✓	?	✓	✓	✓	?	?	✓	?	✓	✓	[6	˟	✓	?
4	✓	✓	✓	✓	✓	˟	˟	?	˟	✓	?	˟	˟	˟	˟	?	˟
5					˟	?	˟			?							
6	?	˟	?	˟	✓	✓	✓	?	˟	✓	?	˟	˟	?	˟	?	˟
7	?	˟	˟	˟	˟	˟	˟	˟	˟	˟	˟	˟	˟	˟	˟	˟	˟
8	?	✓	✓	?	?	✓	?	˟	?	✓	?	˟	˟	?	?	˟	?
9	✓	˟	✓	✓	✓	✓	✓	✓	✓	✓	˟	✓	˟	˟	˟	✓	✓
																	
Results of follow-up^e^																	
Knowledge	n/a	-	/	✓	n/a	n/a	/	/	n/a	/	-	˟	-	n/a	-	-	-
Stigma	n/a	✓˟	˟	˟✓	n/a	n/a	✓✓✓	✓✓	n/a	-	˟	˟	˟	n/a	˟✓	˟˟✓^d^	✓

RCT, randomised controlled trial; Cl-RCT, cluster RCT; NRT, non-RCT; CBA, controlled before-after studies.

a. Intervention type: C, direct contact; I, indirect contact; Ed, education.

b. Duration: 1, one session; 2, >1 session but within 1 week; 3, weekly sessions for 2 weeks or more.

c. Risk of bias: ✓, low risk; ˟, high risk; ?, unclear risk. 1,Was the allocation sequence adequately generated?; 2,Was the allocation sequence adequately concealed?; 3 Were baseline outcome measurements similar (for outcomes included in the review)?; 4,Were baseline characteristics similar?; 5,Were site profiles compared if different sites used as control/intervention?; 6,Was study adequately protected against contamination?; 7,Was knowledge of the allocated interventions adequately prevented during the study (masking)?; 8, Was incomplete outcome data adequately addressed?; 9, Was the study free from selective outcome reporting?

d. Shows the proportion of questionnaire items that did or did not show significant change (i.e. if results in the study are reported by change in individual questionnaire items, or for Opinions about Mental Illness when the six factors are reported separately).

e. Results at follow-up for ‘stigma’ (attitudes, behavioural intentions or affect) and knowledge measures: ✓, statistically significant change in intervention group in stigma-relevant or knowledge outcome measure (summed scores); ˟, no statistically significant difference;-, outcome not measured in study; n/a, outcome not measured in study at this time point. More than one tick or cross in a cell indicates that more than one outcome measure was evaluated. Results from the six outcome measures developed specifically for the interventions they were testing with reliability not measured (or α < 0.7) are not included. If this leaves no results at a time point, this is represented by /.

**Table 2:** Results

	Saporito *et al* (2011)^34^	Economou *et al* (2011)^32^	Pinto-Foltz *et al* (2011)^40^	Chan *et al* (2009)^33^	Pitre *et al* (2007)^28^	Rahman *et al* (1998)^24^	Ventieri *et al* (2011)^31^	Wahl *et al* (2011)^30^	Robinson *et al* (2010)^39^	Naylor *et al* (2009)^29^	Conrad *et al* (2009)^38^	O’Kearney (2009)^27^	O’Kearney *et al* (2006)^26^	Rickwood *et al* (2004)^37^	Schulze *et al* (2003)^36^	Ng & Chan (2002)^35^	Esters *et al* (1998)^25^
Design	RCT	Cl-RCT	Cl-RCT	Cl-RCT	Cl-RCT	Cl-RCT	NRT	NRT	NRT	NRT	? NRT	CBA	CBA	CBA	CBA	CBA	CBA
																	
*Post-test results*																	
Significant effect reported?																	
Knowledge	-	-	˟	✓	-	✓	✓	✓	-	n/a	-	˟	-	✓	-	-	-
Stigma	˟˟	✓✓	˟	✓✓	˟✓^a^	-	✓✓✓	✓✓	✓✓	n/a	✓	˟	˟	✓	˟✓	˟˟✓^a^	✓
Results considered in review^b^																	
Knowledge	-	-	/	✓	-	/	/	/	-	n/a	-	˟	-	/	-	-	-
Stigma	˟˟	✓✓	˟	✓✓	˟✓^a^	-	✓✓✓	✓✓	✓✓	n/a	✓	˟	˟	✓	˟✓	˟˟✓	✓
																	
*Follow-up results*																	
Significant effect reported?																	
Knowledge	n/a	-	✓	✓	n/a	n/a	✓	✓	n/a	˟✓^a^	-	˟	-	n/a	-	-	-
Stigma	n/a	✓˟	˟	˟✓	n/a	n/a	✓✓✓	✓✓	n/a	-	˟	˟	˟	n/a	˟✓	˟˟✓^a^	✓
Results considered in review^b^																	
Knowledge	n/a	-	/	✓	n/a	n/a	/	/	n/a	/	-	˟	-	n/a	-	-	-
Stigma	n/a	✓˟	˟	˟✓	n/a	n/a	✓✓✓	✓✓	n/a	-	˟	˟	˟	n/a	˟✓	˟˟✓^a^	✓

RCT, randomised controlled trial; CI-RCT, cluster RCT; NRT, non-RCT; CBA, controlled before-after studies. ✓, statistically significant change in intervention group in stigma-relevant or knowledge outcome measure (summed scores); ˟, no statistically significant difference; -, outcome not measured in study; n/a, outcome not measured in study at this time point. More than one tick or cross in a cell indicates that more than one outcome measure was evaluated.

a. Shows the proportion of questionnaire items that did or did not show significant change (i.e. if results in the study are reported by change in individual questionnaire items, or for Opinions about Mental Illness when the six factors are reported separately).

b. Do not include results from the six outcome measures developed specifically for the interventions they were testing with reliability not measured (or a50.7). If this leaves no results at a time point, this is represented by /.

Schulze and colleagues summed the amount of positive responses from each student on their novel instrument testing for stereotypes and social distance.^36^ Stereotypes, but not social distance, changed more positively in the intervention than in the control group. This study reported significant differences in baseline outcome measures and baseline characteristics, likely related to the fact their intervention group chose to take part in the mental illness module. Ng & Chan^35^ report a significant improvement in 2/6 Opinion about Mental Illness in Chinese Community (OMICC) factors (benevolence and stigmatisation) between the intervention and control groups, but a significant worsening in both groups in attitudes to restrictiveness. Esters *et al*’s small study (*n* = 40) reported statistically significant positive change on a well-validated scale measuring opinions about mental illness.^25^

#### Studies with positive results at post-test but not follow-up

There were a further four studies that report statistically significant positive results at immediate post-test only (Table 2). They all have high or unclear risk of selection and attrition bias. Pitre *et al*’s three-session puppet show in a primary school reports positive change for the intervention group on the adapted Opinions about Mental Illness (OMI) scale, in 3/6 factors.^28^ Robinson *et al*’s study reports significant changes (compared with baseline and control) after their 2 h session on stigma and attitudes.^39^ The studies of Rickwood *et al*^37^ (one session intervention) and Conrad *et al*^38^ (1-day intervention), do not present any data other than regression statistics, making their findings hard to assess.

#### No positive results

Some studies showed no significant changes at either post-test or follow-up. Saporito *et al*^34^ was the only RCT, randomising at pupil level, although it is not clear what method of randomisation they used. There was no significant improvement in implicit or explicit attitudes to mentally ill people. Pinto-Foltz *et al*^40^ carried out a cluster RCT with more low-risk scores than most of the other studies reviewed. They provided a one-session intervention and found no post-intervention difference in stigmatising attitudes. O’Kearney *et al*’s^26^ and O’Kearney’s^27^ studies of five online sessions (one in males, one in females) recorded results at 5 months. Attitudes (and depression literacy in the later study) were measured as secondary outcomes but showed no significant change.^7^

### Effective intervention design

To answer the second review question it is necessary to see whether there are any consistent features in the intervention programmes in those studies that show positive results. However, the comparison between the results of studies describing such different interventions and methodology is difficult. Chan *et al*^33^ is the only example of a study investigating which aspect of a one-off session might offer the most benefit. The most improvement was seen in the group that had education (a 30 min lecture) followed by a 15 min video (rather than vice versa, or purely education).

Of the studies with positive results at follow-up there is no obvious pattern about what makes a successful intervention. These seven studies include two interventions of only one session and one of the longest interventions (over 10 weeks). Four had no element of contact, two direct, one indirect contact. The follow-up time at which the positive results were recorded ranged from 1 to 12 months. One study was in a primary school.

## Discussion

Within the literature there are frequent references to the existing evidence for the effectiveness of school-based interventions in reducing stigma of mental illness in young people. This systematic review of available evidence does not support those statements. Showing a significant difference in self-report questionnaires immediately after an intervention seems unsurprising and, if that is the limit of the effect of the programme, seems insufficient grounds for rolling out the programme more widely. It is proposed here that a successful programme would show a positive change in outcomes at follow-up, which was the case in seven studies^25,30-33,35,36^ However, the potential for selection and attrition bias, which can exaggerate intervention effect, are common themes in all but one (Economou *et al*^32^) of these studies.

There is one RCT and five cluster RCTs within this body of evidence. Only two of these showed statistically significant improvements in outcome measures at follow-up. Only one of the RCTs clearly described their randomisation process, making it difficult to judge the risk of selection bias in the others. Of the other study designs, Naylor *et al*’s^29^ study stands out as having a greater number of low-risk scores. Small positive changes were seen in their knowledge measure but the validity of the tool used remains doubtful.

There is insufficient data to answer the review question concerning how one might design a successful intervention. Unfortunately, no elements were found to be consistent between the studies with positive results. In the absence of this evidence it is tempting to extrapolate from similar adult studies (summarised in a review as showing positive results^19^). However, two papers present findings that caution against this. ‘In our own voice’ had positive results in adults but ‘disappointing’ results in adolescents^40^ and a more recent meta-analysis of anti-stigma approaches reported that although ‘contact’ was better than education at reducing stigma in adults the reverse was true in adolescents.^21^

Results from studies to date leave uncertainty as to whether interventions to reduce stigma in schools are not effective, whether interventions have been unsuccessful because they have not contained the right combination of elements or whether the studies have not been designed in such a way as to demonstrate efficacy.

Challenges in developing interventions include the need to assess different elements of programme content (contact, educational, etc.) and delivery style against each other. Information is also needed about whether targeting certain groups of children is more successful than universal provision. Indeed, not all students may need an intervention of this type. Only a third of pupils in a Scottish study reported moderate-high levels of stigma.^41^ It is also unclear whether the primary-school age child would be more open to anti-stigma messages, as very few studies have been carried out in this age group.

It is proposed here that the starting assumption when developing an intervention is that it should be long enough and intensive enough to provide some effect at follow-up. The studies reviewed here do not agree on how long a successful intervention should be or at what interval to assess follow-up.

There are daunting issues for study designers to contend with in this field. Stigma is a multifaceted concept, and even well-established measures have their limitations (for example social distance scales not being validated against discriminatory or supportive behaviour^42^). These measures are self-report questionnaires, which are at risk of social desirability bias (particularly, it could be argued, if done after an anti-stigma intervention). The absence of measures to examine change in behaviour after anti-stigma programmes has been recently commented on in a meta-analysis as regrettable.^21^ Maybe resources need to be first directed towards refining age-appropriate measures more closely linked to actual behavioural outcomes. Adverse effects of an intervention also need to be monitored. Recruiting pupils within a school environment is also challenging. Recruitment difficulties in some of the studies described led to a need to actively recruit volunteers to the intervention arm, leading to problems with selection bias. It is also resource heavy to expose the control group to a different type of intervention - hence most of the controls in these studies were simply exposed to ‘normal lessons’.

The protocol of a proposed UK-based, feasibility trial^43^ tackles many of these methodological issues. This well-powered study plans to have an active and randomised control (describing adequate sequence generation and allocation), comparing education with education and contact, carefully prepared material already piloted, 2-week and 6-month follow-up, and will compare the intervention effect by baseline characteristics.^43^ If this trial does not suffer from significant implementation and reporting difficulties the results will be the most definitive to date. This review shows that, although it is inherently attractive to believe that school-based interventions reduce stigma to mental illness in young people, there is currently no strong evidence to support this conclusion.

### Limitations

The limitations of this review include not searching the grey literature and the exclusion of studies written in foreign languages. There is a risk of bias in study selection and data extraction as one author performed these processes. Also, the authors of studies were not contacted for information that could not be gleaned from the published study papers.
